# Increased Plasmatic Levels of Exosomes Are Significantly Related to Relapse Rate in Patients with Oral Squamous Cell Carcinoma: A Cohort Study

**DOI:** 10.3390/cancers15235693

**Published:** 2023-12-02

**Authors:** Samuel Rodríguez-Zorrilla, Alejandro I. Lorenzo-Pouso, Stefano Fais, Maria A. Logozzi, Davide Mizzoni, Rossella Di Raimo, Alessandro Giuliani, Abel García-García, Alba Pérez-Jardón, Karem L. Ortega, Ángel Martínez-González, Mario Pérez-Sayáns

**Affiliations:** 1 Oral Medicine, Oral Surgery and Implantology Unit (MedOralRes), Faculty of Medicine and Dentistry, Universidade de Santiago de Compostela, 15782 Santiago de Compostela, Spain; samuel.rodriguez.zorrilla@outlook.com (S.R.-Z.); abel.garcia@usc.es (A.G.-G.); perlopjm@gmail.com (A.P.-J.); klortega@usp.br (K.L.O.); mario.perez@usc.es (M.P.-S.); 2 ORALRES Group, Health Research Institute of Santiago de Compostela (FIDIS), 15782 Santiago de Compostela, Spain; 3 Department of Oncology and Molecular Medicine, Istituto Superiore di Sanità, Viale Regina Elena 299, 00161 Rome, Italy; stefano.fais@iss.it (S.F.); mariantonia.logozzi@iss.it (M.A.L.); 4 ExoLab Italia, Tecnopolo d’Abruzzo, 67100 L’Aquila, Italy; davide.mizzoni@iss.it (D.M.); rossella@exolabitalia.com (R.D.R.); 5 Department of Environment and Health, Istituto Superiore di Sanità, Viale Regina Elena 299, 00161 Rome, Italy; alessandro.giuliani@iss.it; 6 School of Dentistry, Department of Oral Pathology, University of São Paulo, Av. Lineu Prestes, 2227, Cidade Universitária São Paulo, Sao Paulo 05508-000, Brazil; 7 Endocrinology and Nutrition Service, Complejo Hospitalario Universitario de Pontevedra, Mourente S/N, 36472 Pontevedra, Spain; angelmg88@hotmail.com; 8 Institute of Materials (IMATUS), Avenida do Mestre Mateo, 25, 15782 Santiago de Compostela, Spain

**Keywords:** NTA, liquid biopsy, plasmatic exosomes, mouth neoplasm, screening test

## Abstract

**Simple Summary:**

This study investigates the potential of exosomal profiles as a tool for early detection of relapse and long-term outcomes in oral squamous cell carcinoma (OSCC) patients undergoing conventional therapy. Examining 27 OSCC patients, this study found a significant reduction in presurgical exosome levels after surgery. Patients experiencing relapse showed higher postsurgical exosome concentrations and larger sizes compared to disease-free individuals. Lower presurgical exosome levels were correlated with better disease-free survival. Receiver operating characteristic analysis indicated the promising diagnostic efficacy of presurgical exosome concentrations in identifying relapse (AUC = 0.82). The findings suggest that presurgical exosomal plasma levels independently predict early recurrence and survival in OSCC, proposing peripheral exosome detection as a novel tool for clinical management with implications for prognosis assessment.

**Abstract:**

Background: Oral squamous cell carcinoma (OSCC) is characterized by an immunosuppressive tumor microenvironment. Their plasma-derived exosomes deliver immunomodulatory molecules and cargo that correlate significantly with clinical parameters. This study aims to assess the exosomal profile as a potential tool for early detection of relapse and long-term outcomes in OSCC patients undergoing conventional therapy. Methods: 27 OSCC patients with a median 38-month follow-up were included in this study. The relationship between NTA-derived parameters and clinical pathological parameters was examined, and receiver operating characteristic (ROC) curves were utilized to evaluate the diagnostic efficacy of these values in detecting cancer relapse. Results: Plasmatic levels of exosomes prior to surgery showed a drastic reduction after surgical intervention (8.08E vs. 1.41 × 10^9^ particles/mL, *p* = 0.006). Postsurgical concentrations of exosomes were higher in patients who experienced relapse compared to those who remained disease-free (2.97 × 10^9^ vs. 1.11 × 10^9^ particles/mL, *p* = 0.046). Additionally, patients who relapsed exhibited larger exosome sizes after surgery (141.47 vs. 132.31 nm, *p* = 0.03). Patients with lower concentrations of exosomes prior to surgery demonstrated better disease-free survival compared to those with higher levels (*p* = 0.012). ROC analysis revealed an area under the curve of 0.82 for presurgical exosome concentration in identifying relapse. Conclusions: Presurgical exosomal plasmatic levels serve as independent predictors of early recurrence and survival in OSCC. All in all, our findings indicate that the detection of peripheral exosomes represents a novel tool for the clinical management of OSCC, with potential implications for prognosis assessment.

## 1. Introduction

Oral squamous cell carcinoma (OSCC) is an aggressive form of cancer that represents a growing public health problem, globally accounting for 377,713 new cases and 48,143 deaths annually [1]. Even with relevant progress in surgical treatment, chemoradiotherapy, radiotherapy, and immunotherapy, the OSCC patients’ overall survival remains low as compared to other tumors [2,3]. Risk factors for OSCC are broadly classified into three categories: chemical, physical, and viral mechanisms [4]. However, from a molecular perspective, OSCC exhibits several hallmarks of cancer, such as sustained proliferative signaling, evasion of growth suppressors, resistance to cell death, and the ability to induce angiogenesis, invasion, and metastasis. Additionally, OSCC cells exhibit deregulated cellular energetics, a high level of immune escape associated with tumor-promoting inflammation, and rates of both genome instability and mutations [5,6].

Tissue biopsy is the gold standard for the diagnosis of oral cancer as well as all solid tumors. Liquid biopsies have the potential to help in identifying molecular features related to tumor behavior, such as invasive capacity, relapse rate, and metastasis. This technique is being used with increasing force in other types of cancers of the digestive system [7]. Until a decade ago, liquid biopsy was confined to circulating tumor cells and nucleic acids, either DNA or RNA. Although today they continue to be useful for the prognosis of oral squamous cell carcinoma [8]. Circulating exosomes are emerging as a very promising new noninvasive tool for the clinical management of tumor patients [9,10,11].

Exosomes are nanosized extracellular vehicles having origins in the re-cycling of intracellular vesicles. They have demonstrated that they exert a key role in intercellular communication in both physiological and pathological scenarios [12,13,14,15]. Exosomes are the natural cargo vesicles for proteins, DNAs, RNAs, lipids, and metabolites that are essential in a myriad of physiological and pathological functions [16,17]. Exosomes play a Janus-faced role in head and neck cancers [18,19,20,21]. It has been shown that exosomes can deliver tumor-associated antigens to dendritic cells, inducing anticancer immunity, preserving homeostasis, and playing cytoprotective roles [22]. Conversely, recent clinical data have shown that a distinguishing and relevant hallmark of cancer patients is the increased plasmatic exosome levels that are not tumor-specific, being common in many cancers but distinguishing cancer from a healthy condition [23,24,25]. Exosome cargo is primarily involved in tumor progression and metastasis [26,27]. Exosomes are also involved in multidrug resistance, being responsible for the extracellular elimination of chemotherapeutics [28,29,30] in the epithelial–mesenchymal transition and in extracellular matrix remodeling [31]. On the other hand, and as a consequence of the aforementioned, it is crucial to highlight the role of exosomes as an oral delivery vehicle for cancer therapies. Various reviews debate and analyze strategies in which the use of exosomes has yielded encouraging results in exploring diagnostic and treatment options for various types of cancer in humans [32]. Notably, tumor exosomes have a key role in the development of metastasis in both setting a sort of “premetastatic niche” and in transformingmesenchymal stem cells (MSC), contained in target organs, into tumor-like cells [33,34]. Similarly, in the opposite direction, recent studies have indicated a crucial involvement of exosomes derived from MSC-derived exosomes in conferring resistance to chemotherapy agents, targeted therapy drugs, radiotherapy, and immunotherapy in cancer [35,36]. Therefore, the presence of a high level of tumor exosomes in the plasma of tumor patients is a sign that predicts the development of metastasis.

Recently, a pilot clinical study from our group showed different levels of plasmatic exosomes in patients with OSCC before and after surgical treatment. In fact, our study showed a dramatic reduction in the plasmatic levels of exosomes expressing CD63 and of the surrogate tumor marker Caveolin-1 (cav-1) after ablative surgery [23]. This previous study used a pioneering method to analyze and characterize exosomes by means of immunocapture-based ELISA previously reported by our group and further validated by benchmarking against two existing gold standards, namely nanoparticle tracking analysis (NTA) and nanoscale flow cytometry [23,37], and summarized it as a technique in a recent article [38]. The potential use of exosomes as a natural delivery for anticancer molecules is under investigation [15,25]. However, much still needs to be understood about the source of exosomes to deliver therapeutic molecules. The use of human exosomes has raised many problems, and at the moment, they are not allowed for clinical use. An interesting new approach could be the use of plant-derived exosomes, particularly those derived from organic agriculture, that are entirely free of toxic molecules [39]. Moreover, very recently, the ability of plant-derived exosomes to exert clear antitumor activity has been shown [40]. The ensemble of these reports highly supports the revolutionary role of exosomes in the clinical management of tumor patients.

Building upon these preliminary results, we embarked on a prospective cohort study aiming to assess the clinical relevance of plasmatic exosome number and size distribution using NTA in a larger cohort with an extended follow-up period to refine our estimates.

## 2. Materials and Methods

### 2.1. Study Design

This study was a prospective cohort study aimed at assessing the population of exosomes before and after surgery and their correlation with the long-term outcomes of patients. The study protocol was approved by the Clinical Research Ethics Committee of Galicia under ethical code 2018/435, and it was designed in accordance with the STROBE recommendations [41]. All procedures were conducted in accordance with the Helsinki Declaration and its subsequent modifications, with the full understanding and written consent of all participants. No identifying data were recorded to ensure the anonymity of participants.

### 2.2. Patient Selection and Clinical Data

This study included 27 subjects who were prospectively recruited and diagnosed with OSCC. The inclusion criteria were as follows: patients over the age of 18, of both genders, diagnosed with OSCC in stages T1–T4, and who underwent tumor resection and lymph node resection surgery if necessary. The follow-up period started in 2014 and lasted until mid-2022. The exclusion criteria consisted of patients with tumors of any other origin in any part of their body, immunological diseases, a previous history of radiotherapy or chemotherapy treatment, or those using proton pump inhibitors or any other antacid drug.

Each patient underwent a comprehensive clinical examination, including a medical history assessment, detailed clinical assessment, tumor biopsy, complementary radiology techniques, and sentinel lymph node evaluation. Staging was performed according to the 8th edition of the *American Joint Committee on Cancer’s Cancer Staging Manual*. The established protocol of the University Hospital of Santiago de Compostela (Santiago de Compostela, Corunna, Spain) was followed [41,42]. The decision to perform surgery was made by the maxillofacial surgeon based on a comprehensive examination and deliberation by the CHUS committee for tumors.

Patients were followed up for an average of 38.56 ± 23.36 months. During this period, data on clinicopathological features (i.e., tumor location, TNM stating, T status, N status, presence of local metastasis, and tumor differentiation), relapse, overall survival (OS), and disease-free survival (DFS) were collected.

### 2.3. Sample Collection and Plasmatic Exosomes Characterization and Quantification

Before surgery, two plasma tubes of 1 mL each were obtained from each patient and stored at −80 degrees Celsius until transfer to the molecular oncology laboratory of the Istituto Superiore di Sanità (Rome, Italy). The same procedure was repeated 7 days after surgery [21].

To obtain plasma from blood samples, EDTA-treated blood from patients was centrifuged at 400× *g* for 20 min. Plasma was collected and stored at −80 °C until analysis. Upon thawing, 1 mL of plasma underwent a centrifugal procedure to remove cell debris, organelles, microvesicles, and pellet exosomes. In the final step, plasma samples were centrifuged for 1 h and 30 min at 110,000× *g* using a Fiberlite™ F50L-24 × 1.5 fixed-angle rotor, K-Factor: 33 (Thermo Fisher Scientific, Waltham, MA, USA) in the Sorvall WX Ultracentrifuge Series (Thermo Fisher Scientific) to obtain the exosomal pellet, which was then washed in PBS and resuspended in an appropriate buffer for subsequent analysis. Specifically, the exosomal pellet was resuspended in PBS forNTA.

NTA from Malvern (NanoSight NS300, Worcestershire, UK) was used to measure the size distribution and concentration of exosome samples in liquid suspension in the range of 10–1000 nm based on the analysis of Brownian motion [43]. After laser beam illumination, light scattering allowed for the visualization, recording, and tracking of particles with a CCD or CMOS camera. Five videos of typically 60 s duration were taken. Data were analyzed using the NTA 3.0 software (Malvern Instruments, Malvern, UK), which was optimized to detect and track each particle on a frame-by-frame basis. NTA is based on the phenomenon of the random movement (diffusion) of small particles when dispersed in a liquid, enabling direct and precise measurement of particle concentration and size. The Brownian motion of each particle was tracked using the Stokes–Einstein equation: D° = kT/6πηr, where D° is the diffusion coefficient, kT/6πηr = f0 is the frictional coefficient of the particle, for the special case of a spherical particle of radius r moving with a uniform velocity in a continuous fluid of viscosity η, k is Boltzmann’s constant, and T is the absolute temperature.

The evaluation of the particle size distribution (PSD) was performed using the parameters mean, mode, and standard deviation (SD), which indicate the average, most frequent particle class size, and variability of the analyzed particles, respectively. The same statistical descriptors were adopted to summarize the number of particle distributions.

### 2.4. Statistical Analysis

The statistical analysis was performed using patients affected by OSCC as basic units. Data were analyzed using IBM SPSS Statistics 20.4 (SPSS Inc., Chicago, IL, USA) and R v.4.1.0 software. Descriptive statistics were reported for each variable based on its distribution, determined by the Shapiro–Wilk test. Gaussian distributed variables were reported as mean ± SD with 95% confidence intervals (CIs), while continuous non-normally distributed variables were reported as median (interquartile range). Categorical, normally distributed variables were reported as percentages.

Given the inherent pairing of the data, involving observations before and after surgery, inferential tests were conducted in their corresponding mode. A paired *t*-test (or equivalently, a Mann–Whitney or Chi-square test) was used to compare pre- and postsurgery measurements. Univariate survival analysis was conducted using Kaplan–Meier and log-rank tests for both OS and DFS, which were used to compute hazard ratios (HRs). Multinomial logistic regression models were constructed to determine the risk of relapse based on exosome quantification and characterization. The cutoff point for classifying patients as having a high or low concentration and size of exosomes was based on the arithmetic mean of these values, thus considering these values per patient as over or underexpressed.

The best discriminant cut-off thresholds of the mean presurgical and postsurgical exosomal concentrations, as well as their difference, to predict OSCC relapse, were computed using a receiver operating characteristic (ROC) curve. A bootstrap resampling method (k = 1000) was used for output. The criterion chosen for model selection was the Akaike information criterion to limit the possibility of collinearity effects [44]. A logarithmic transformation of exosome concentrations was used for these analyses due to the violation of normality in the residuals of the regression model using raw data at the lower and upper tails.

To address the possibility of residual confounding by age on presurgical exosome concentration and PSD, a Pearson correlation was performed separately for cases with and without cancer recurrence. No statistically significant correlations were found (Figure S1). A *p*-value of 0.05 was considered statistically significant for all tests.

## 3. Results

### 3.1. Sample Description

The sample for this study consisted of 27 patients, with 14 males and 13 females. The mean age of the patients was 70.35 (15.79) years. All the tumors included in this study were from the oral cavity, with six located in the gingiva, five in the tongue, and six in the hard palate, which were the most frequent locations. Among the patients, 11 were smokers, with men smoking an average of 18 ± 16.7 cigarettes compared to women who smoked an average of 5.3 ± 9.7 cigarettes (*p* = 0.024). In terms of tumor stage, 15 patients were diagnosed in the initial stages (I and II), while 12 patients were diagnosed in the advanced stages (III and IV). Most of the tumors (*n* = 14) exhibited moderate differentiation. For detailed information, refer to Table 1.

### 3.2. Concentration and Size Distribution of Plasmatic Exosomes

The concentration of exosomes was evaluated both presurgically and after oncological surgery. The mean presurgical exosome concentration was 1.02 × 10^9^ ± 5.89 × 10^8^ particles/mL, ranging from 1 × 10^8^ to 2 × 10^9^ particles/mL. The mean postsurgical exosome concentration was 1.76 × 10^9^ ± 1.98 × 10^9^ particles/mL, ranging from 3 × 10^8^ to 9 × 10^9^ particles/mL. Regarding exosome size, the presurgical distribution had a mean of 133.57 ± 17.46 nm, ranging from 107.1 to 171.7 nm. The postsurgical distribution had a mean of 125.52 ± 10.36 nm, ranging from 111.2 to 154 nm.

**Figure 1:**
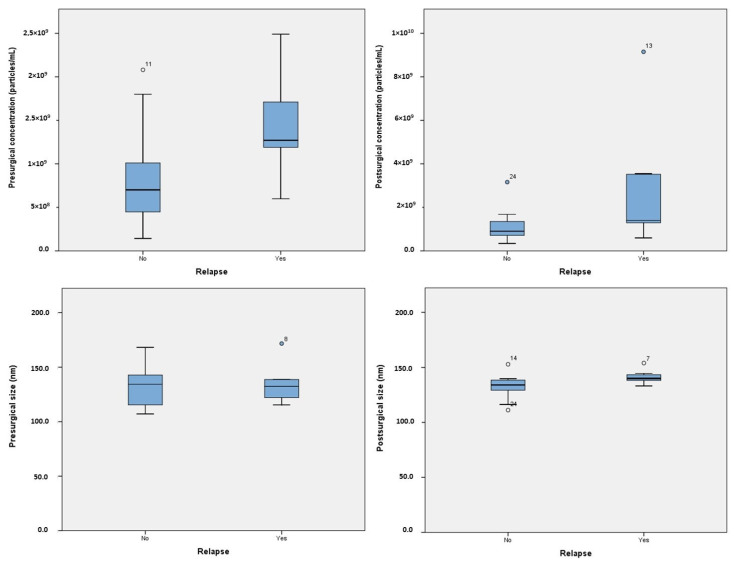
Boxplot representing the exosome levels and sizes, both presurgical and postsurgical.

While there was a positive correlation between pre- and postsurgical concentrations, the correlation did not reach statistical significance (r = 0.398; *p* = 0.091). In the overall analysis, no significant differences were observed in the concentrations (*p* = 0.152) or dimensions (*p* = 0.933) of exosomes before and after surgery (Figure 1). However, when analyzing the sample based on relapse, it was observed that patients who experienced relapse had higher concentrations of exosomes presurgically but a significant reduction in exosome number after surgery (8.08 × 10^8^ [95%CI 5.52 × 10^8^–1.06 × 10^9^] vs. 1.41 × 10^9^ [95%CI 9.77 × 10^8^–1.85 × 10^9^] particles/mL, *p* = 0.006) (Table S1). 

**Figure 2:**
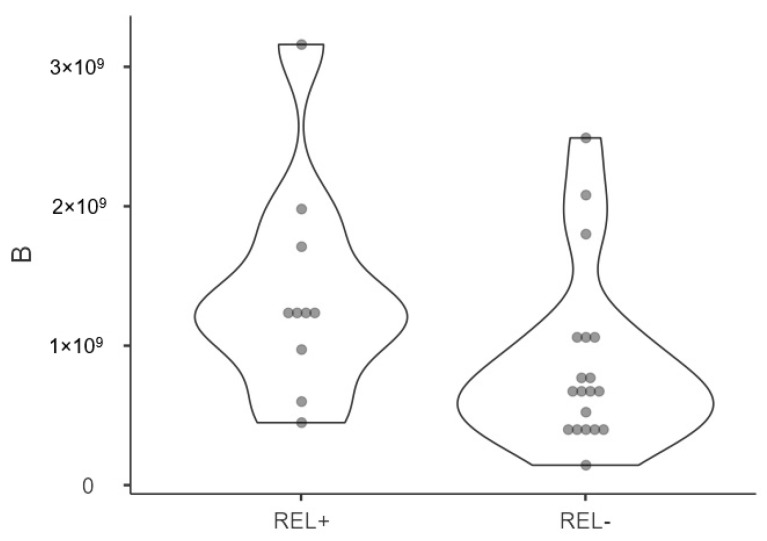
Violin plot displaying NTA distribution and quantification of patients affected by relapse (REL+) and not (REL-) of presurgical plasmatic exosome concentrations (nm) showing density probability with kernel smoothing.

A violin plot illustrating the distribution of presurgical exosome concentration in patients with and without relapse is presented in Figure 2. Postsurgical concentrations were statistically higher in patients who experienced relapse (2.97 × 10^9^ [95%CI 2.3 × 10^8^–5.7 × 10^8^] vs. 1.11 × 10^9^ [95%CI 6.66 × 10^8^–1.55 × 10^9^] particles/mL, *p* = 0.046). In terms of dimensions, patients who experienced relapse had larger postsurgical exosome sizes (141.47 nm [95%CI 135.39–147.55] vs. 132.31 nm [95%CI 125.78–138.84], *p* = 0.03). No significant differences in exosomal concentration or size were found based on gender, tumor location, or tobacco use.

### 3.3. Survival Analysis

**Figure 3:**
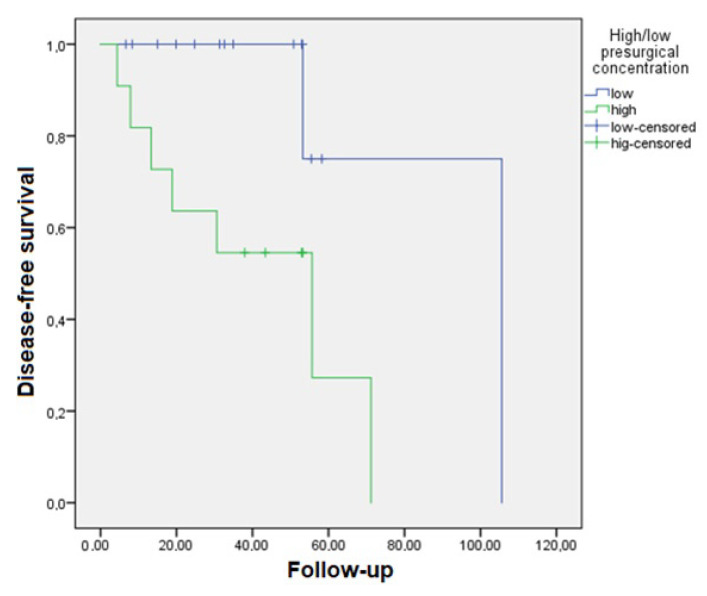
Kaplan–Meier curve for disease-free survival in patients with a lower presurgical plasmatic exosome concentration (particles/mL) who survived longer than those with higher concentrations (*p* = 0.012).

The mean follow-up time for this study was 38.56 ± 23.26 months. DFS was 26.44 ± 26.46 months, while the OS was 18.22 ± 11.71 months. Kaplan–Meier survival models were constructed for DFS and OS (Table S2). Patients with low concentrations of exosomes prior to surgery had a significantly higher DFS (92.49 [95%CI 61.06–123.93] months) compared to patients with high levels (41.42 [95%CI 24.31–58.53] months, *p* = 0.012). Refer to Figure 3 for the corresponding survival curves.

### 3.4. Relapse Analysis

**Figure 4:**
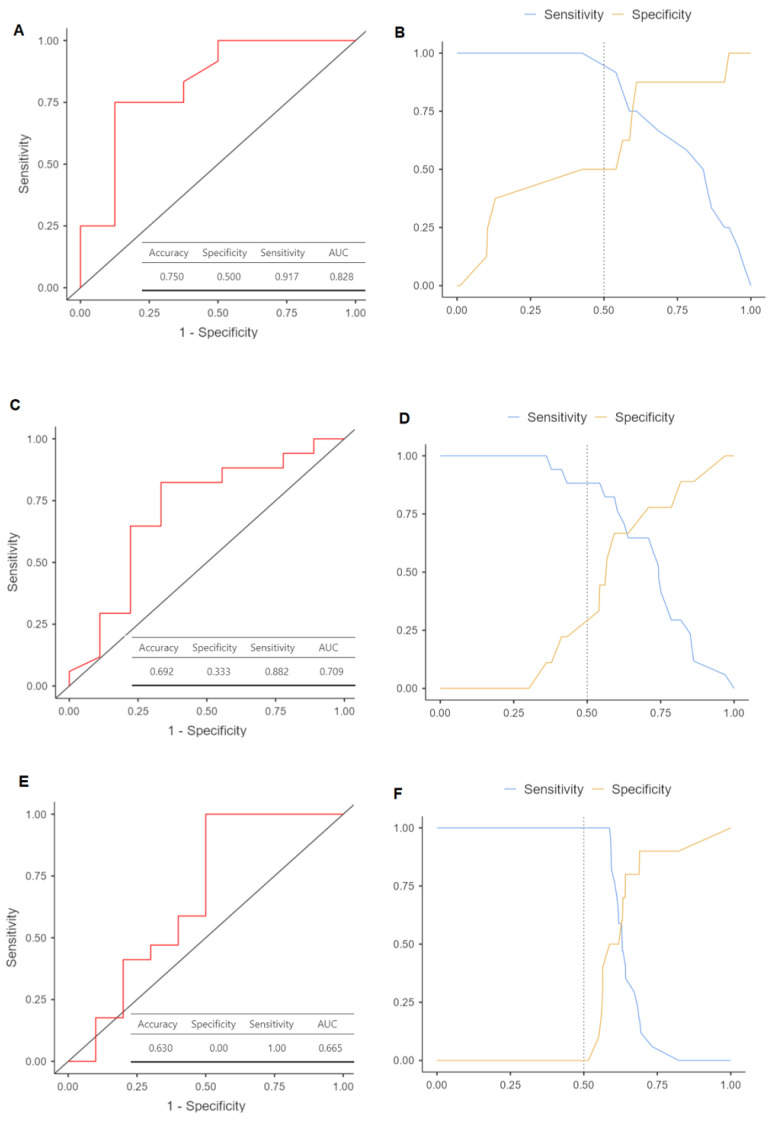
Receiver operating characteristic (ROC) curves to predict oral squamous cell carcinoma relapse based on log10 levels of plasmatic exosome concentrations: (**A**) ROC plot with its area under the curve (AUC) for presurgical exosomes (*p* = 0.012); (**B**) cut-off plot representing the intersection that allows maximizing the level of sensitivity and specificity of the model used; (**C**) ROC plot with its AUC for postsurgical exosomes (*p* = 0.035); (**D**) cut-off plot representing the sensitivity and specificity of the model; (**E**) ROC plot with its AUC for the difference of concentrations between pre- and postsurgial exosomes (*p* = 0.043); (**F**) cut-off plot representing the sensitivity and specificity of the model.

Multinomial logistic regression models were constructed to assess the risk of recurrence associated with different parameters of plasmatic exosomes and clinicopathological variables. It should be noted that the tumor stage and related variables did not yield the most optimal models. Despite the direct association between stage as N status and DFS and OS, incorporating these variables into the models did not enhance the estimations for the compute. The most accurate models included adjustments for gender (Table 2). The results indicated that patients with elevated presurgical plasma levels of exosomes had increased risk of recurrence, both in the univariate model (HR = 11.37 [95%CI 1.65–78.37], *p* = 0.014) and the adjusted model (HR = 18.35 [95%CI 1.61–208.35], *p* = 0.019). The minimum and maximum log10 exosome concentration values, both before and after surgery, and their differences were analyzed to establish an approximate range for patients with relapse compared to those who remained disease-free at the end of the follow-up period. ROC analysis for presurgical exosome concentration yielded an area under the curve (AUC) of 0.82 for identifying OSCC relapse (Figure 4A), with a sensitivity of 50.0% and a specificity of 91.7% (Figure 4B). For postsurgical concentration, the AUC was 0.79 (Figure 4C), with a sensitivity of 33.3% and a specificity of 88.2% (Figure 4D). The AUC for the difference in exosome concentrations before and after surgery was 0.665 (Figure 4E), with a sensitivity of 0% and a specificity of 100% (Figure 4F).

## 4. Discussion

In a comprehensive study involving 27 patients, the concentration and size distribution of plasmatic exosomes in OSCC were extensively explored. Through meticulous quantitative analysis, the mean presurgical exosome concentration was identified as 1.02 × 10^9^ ± 5.89 × 10^8^ particles/mL, while the postsurgical concentration averaged 1.76 × 10^9^ ± 1.98 × 10^9^ particles/mL. Intriguingly, a positive correlation was observed between pre- and postsurgical concentrations, with a significant reduction in exosome number after surgery for patients experiencing relapse. Survival analysis revealed compelling results, indicating a substantial impact of exosome concentrations on disease-free and overall survival. These findings not only contribute valuable insights into OSCC prognosis but also underscore the potential significance of exosome concentrations in predicting relapse, offering avenues for further exploration in cancer research.

The current gold standard for early detection of OSCC is tissue biopsy and subsequent histopathological analysis. Moreover, tumor staging classification according to the TNM system is mandatory for treatment decision-making and prognosis assessment [45,46]. However, it lacks the ability to reliably predict the biological characteristics of tumors, rendering it of minimal significance when determining treatment approaches influenced by the tumor’s biological behavior [47].

The trigger for this study stemmed from the observation that, despite the increasing number of potentially useful biomarkers for OSCC, significant challenges remain in their clinical translation [48,49]. Traditionally, tissue-based biomarkers, particularly immuno-histochemical analysis, have dominated this field [50,51,52].

This study contributes to the growing body of evidence supporting the utility of exosomes as new noninvasive diagnostic/prognostic tools for OSCC [53,54,55,56,57]. In the case of head and neck cancer, Bergmann et al. were the first to reveal the capacity of serum exosomes to induce T-cell apoptosis, linking this molecular machinery with poor locoregional control and the presence of distant metastases [58]. Exosomes in liquid biopsies capture the dynamic landscape of oral neoplasms, making them potential vehicles for OSCC biomarkers [59]. Additionally, in the context of these solid neoplasms, exosome cargo has proven abilities to create a particular hypoxic tumor microenvironment, enhancing tumor aggressiveness and metastasic potential [60].

Here, through the implementation ofNTA, plasmatic exosome ability proved to be linked with the survival and clinicopathological characteristics of OSCC patients. A lack of correlation in terms of age between subgroups was preliminarily demonstrated [61]. Consequently, the data were analyzed to stratify patients based on both the number and size of peripheral exosomes and their clinicopathological and long-term outcomes.

This investigation has revealed a noteworthy association between pre- and postsurgical exosome levels and a statistically significant risk of disease recurrence, as previously reported [62,63,64,65]. Interestingly, a study comparing disease-free intervals in ovarian cancer patients reported a negative correlation with cav-1 exosomes, revealing a paradoxical relationship between exosome expression and cancer prognosis [66]. Jiang et al. have recently confirmed these phenomena in liver tumors [67]. On the other hand, multiple studies support the notion that the surrogate marker used in our pilot study, i.e., cav-1 overexpression, is directly related to disease progression and has an outstanding predictive ability to stratify affected patients in terms of prognosis, distinguishing it from other tumor types [68,69]. Moreover, a correlation between patient survival and the number of exosomes prior to surgery was ascertained, consistent with our preliminary study. Other studies have attempted to investigate the exosome cargo related to this nexus in OSCC. He et al. demonstrated the greatest power of a reduced concentration of plasmatic exosomes prior to surgery in predicting the absence of recurrence through microRNA-130a exosome cargo [70]. Deng et al., examining peripheral concentrations of exosome hsa_circRNA_047733 in OSCC, created a dynamic nomogram integrating five other clinical variables to predict recurrence with a powerful diagnostic yield (AUC = 0.87) [71]. On the other hand, several in vitro and in vivo reports have described various molecular pathways related to the effect of exosomes in OSCC, such as the p38, Akt, SAPK/JNK, Wnt/β-catenin, or ERK1/2 signaling pathways [72,73,74]. Moreover, in vitro research on OSCC cell lines has shown the ability of certain exosome cargo to disrupt the proper function of some cells. In this vein, Wang et al. showed that exosomal NAP1 enhances the cytotoxicity of natural killer cells [75]. Pan et al. showed that oral cancer cell-secreted exosomal CMTM6 induced M2-like macrophage polarization as PD-L1 expression [74].

Regarding exosome size, measured in nanometers, our findings have revealed a significant correlation between larger exosome size following surgical intervention and disease recurrence in affected patients. This observation is particularly intriguing given the growing body of literature highlighting the role of exosome content in promoting tumor progression through diverse mechanisms [76,77,78]. In this vein, Deng et al. demonstrated an association between STIM1, a protein involved in tumor angiogenesis, and larger EBV-LMP1-containing exosomes using nasopharyngeal carcinoma as a model [74].

The relapse analysis showed a more than 10 times greater probability of disease recurrence in those patients with a higher concentration of exosomes prior to surgery. ROC curve analysis, particularly for exosome concentration prior to surgery, demonstrated a significant discriminative value in this study, with an AUC of 0.82, a sensitivity of 50.0%, and a specificity of 91.7%. Tests examining diagnostic yield confirmed that the measurement of presurgical exosome concentration was an effective method for prospectively distinguishing between OSCC patients affected by relapse or not after conventional treatment. This corroborates data from a previous study by Li et al., although in our case, a superior AUC was obtained, probably due to the exosome analysis being built from a different pipeline based on ELISA analysis [79].

He et al. obtained similar results when investigating plasma-derived exosome microRNA-130a, inferring through a multivariate analysis that patients with high levels of these extracellular vesicles had a value nearly threefold higher than those with low levels in terms of overall survival [70]. Ju et al. also verified that the relapse-free survival time of tongue carcinoma patients with low and high expression of exosomes derived from LINC00152 was significantly higher in patients with a lower exosome concentration [80]. This study significantly contributes to the clinical application of plasma exosome measurements in the follow-up of OSCC patients. Moreover, this prospective clinical study is the first one in OSCC comparing the plasma exosome levels before and after surgery, providing a significant relationship with exosomes both before and after surgery and relapse risk, time free of disease, and life expectancy of patients for nearly 5 years. An interesting finding was that exosome parameters returned to normal shortly after ablative surgery, aligning with a previous effort of our group related to Hsp60-related exosomes in bowel cancer [81]. The data presented herein encourages the belief that exosome concentration is a candidate for theranostics in OSCC.

Based on clinical evidence, it was observed that variation in exosome levels represents a tumor biomarker that enables the prediction of patient outcomes, mandatorily needing closer follow-up in patients with higher plasmatic levels [82,83]. Furthermore, the detection of a possible recurrence can be identified in a timely manner. The term “circulating tumor mass”, previously discussed in the pilot article, is becoming increasingly relevant due to its practical association with disease prognosis [84,85,86]. Previous studies have correlated the increased plasmatic levels of exosomes in tumor patients to the acidic microenvironment [37,87,88].

As limitations of our study and in acknowledging potential confounding issues related to cancer progression, metastases, and therapeutic interventions, we believe in the need to conduct broader studies in the future. However, due to the scale of our study, there is a risk of overfitting or limited stratification. In this context, the identified confounding factors may diminish the already limited statistical power of this study, steering the results in a conservative direction and increasing the difficulty of achieving significance. It is crucial to recognize that, in a naturalistic setting, a prognostic marker must withstand variations in contextual conditions.

In summary, the findings from this study have great potential in cancer clinics by implementing current clinical indicators of tumor progression such as tumor size, overall survival, and disease-free interval. From these results, it appears conceivable that measuring exosome plasmatic levels before tumor resection may help in determining the best surgical approach.

## 5. Conclusions

This study sheds light on the potential use of exosomes as a noninvasive biomarker for OSCC patients. Surgical treatment was found to induce a significant reduction in plasma levels of exosomes, and high concentrations of exosomes were associated with an increased risk of disease recurrence. On the other hand, patients with low concentrations of exosomes prior to surgery demonstrated better survival.

These findings have important clinical implications, as they provide a potential new strategy for improving the clinical follow-up of OSCC patients. The noninvasive nature of exosome quantification and characterization makes it a valuable tool for monitoring disease progression and detecting recurrence in a timely manner, thus hopefully improving patient prognosis. However, further studies are needed to confirm the results of this study and explore the potential clinical applications of exosomes as a tumor biomarker for OSCC. This study emphasizes the importance of characterizing and quantifying exosomes in tumor patients’ body fluids, most of all in the plasma [89]. Of course, larger multicenter clinical studies are mandatory in order to validate the results of this study in a larger number of patients.

## Figures and Tables

**Table 1:** Characteristics of the studied population.

Variables		*n* (%)
Qualitative Analysis		
Gender	Male	14 (51.9)
	Female	13 (48.1)
Tumor localization	Buccal mucosa	3 (11.1)
	Gum	6 (22.2)
	Floor of the mouth	3 (11.1)
	Retromolar trigone	3 (11.1)
	Hard palate	5 (18.5)
	Tongue	5 (18.5)
	Maxilla	2 (7.4)
Tobacco consumption	No	14 (51.9)
	Yes	11 (40.7)
	Ex consumer	2 (7.4)
Tumor stage	Stage 1	8 (29.6)
	Stage 2	7 (25.9)
	Stage 3	2 (7.4)
	Stage 4	10 (37)
TNM stage (T)	T1	7 (25.9)
	T2	7 (25.9)
	T3	3 (11.1)
	T4	9 (33.3)
TNM stage (N)	N1	3 (11.1)
	N2	9 (33.3)
	N3	1 (3.7)
TNM stage (M)	M0	24 (88.9)
	M1	3 (11.1)
Differentiation	Mild	12 (44.4)
	Moderate	14 (51.9)
	Severe	1 (3.7)
Radiotherapy	No	17 (63)
	Yes	10 (37)
Chemotherapy	No	22 (81.5)
	Yes	5 (18.5)
Local metastasis	No	24 (88.9)
	Yes	3 (11.1)
Relapse	No	18 (66.7)
	Yes	9 (33.3)
Exitus	No	17 (63)
	Yes	10 (37)
Quantitative analysis	**Mean ± SD**	**Min–max**
Age	70.35 (13.57)	46.7–100.19
Cigarettes per day	11.89 (14.96)	0–40
Presurgical concentration (particles/mL)	1.02 × 10^9^ (5.89 × 10^8^)	1 × 10^8^–2 × 10^9^
Postsurgical concentration (particles/mL)	1.76 × 10^9^ (1.98 × 10^9^)	3 × 10^8^–9 × 10^9^
Presurgical (nm)	133.57 (17.46)	107.1–171.7
Postsurgical (nm)	135.52 (10.36)	111.2–154
Follow-up (months)	38.56 (23.36)	4.37–105.59
Follow-up with relapse patients (months)	26.46 (26.44)	2.43–85.37
Follow-up with exitus patients (months)	18.22 (11.71)	4.37–34.92

SD, standard deviation.

**Table 2:** Relapse risk according to low/high concentrations of exosomes.

Variable	Univariate (HR 95%CI)	*p*	Adjusted (HR 95%CI)	*p*-Value
Presurgical concentration				
High vs. low	11.37 (1.65–78.37)	**0.014**	18.35 (1.61–208.35)	**0.019**
Male vs. female	1.56 (0.31–7.82)	0.587	0.38 (0.04–4.22)	**0.433**
Postsurgical concentration				
Particles/mL				
High vs. low	9.00 (0.717–113.016)	0.89	9.00 (0.660–122.794)	0.99
Male vs. female	1.56 (0.31–7.82)	0.587	1.00 (0.124–8.090)	1
Presurgical dimension (nm)				
High vs. low	2.25 (0.419–12.092)	0.345	2.17 (0.398–11.897)	0.370
Male vs. female	1.56 (0.31–7.82)	0.587	1.261 (0.239–6.659)	0.785
Postsurgical dimension (nm)				
High vs. low	1.200 (0.185–7.770)	0.848	1.337 (0.194–9.202)	0.768
Male vs. female	1.56 (0.31–7.82)	0.587	1.653 (0.246–11.088)	0.605

CI, confidential interval; HR, hazard ratio. Significant results are reported in bold.

## Data Availability

The data will be shared by requesting the first author.

## Supplementary Materials

The following supporting information can be downloaded at: https://www.mdpi.com/article/10.3390/cancers15235693/s1, Figure S1: Correlation between age (years) and A serum exosomes concentration (particles/mL); B exosomal size (nm); Table S1: Results in relapse and non-relapse patients; Table S2: Survival models by Kaplan Meier for disease free survival (DFS) and overall survival (OS).
