# Supplementary material for: Increased Plasmatic Levels of Exosomes Are Significantly Related to Relapse Rate in Patients with Oral Squamous Cell Carcinoma: A Cohort Study
Source: Cancers (Basel). 2023 Dec 2;15(23):5693. doi: 10.3390/cancers15235693 (PMC10705147; doi:10.3390/cancers15235693)

Table S1. Results in relapse and non-relapse patients.

Variable		Mean ± SD	95%CI	p (t-test)
**Presurgical concentration (particles/mL)**	No relapse	8.08E+8 ± 4.98E+8	5.52E+8 to 1.06E+9	**0.006**
	Relapse	1.41E+9 ± 5.65E+8	9.77E+8 to 1.85E+9	
**Pos surgical concentration (particles/mL)**	No relapse	1.11E+9 (7.34E+8	6.66E+8 to 1.55E+9	**0.046**
	Relapse	2.97E+9 ± 2.96E+9	2.3E+8 to 5.7E+8	
**Presurgical (nm)**	No relapse	134.15 ± 18.17	124.80 to 143.49	0.916
	Relapse	132.47 ± 17.03	119.38 to 145.57	
**Pos surgical (nm)**	No relapse	132.31 ± 10.8	125.78 to 138.84	**0.03**
	Relapse	141.47 ± 6.57	135.39 to 147.55	
**Age**	No relapse	71.55 ± 14.47	64.35 to 78.75	0.668
	Relapse	67.94 ± 11.99	58.73 to 77.16	

CI, confidential Interval; SD, standard deviation. Significant results are reported in bold.

Table S2. Survival models by Kaplan Meier for disease free survival (DFS) and overall survival (OS).

Variable		Estimated average	95%CI	p (long-rank test)	Estimated average	95%CI	p (long-rank test)
		OS			DFS		
**Presurgical concentration**	**Low**	**73.56**	**51.20-95.91**	**0.549**	92.49	61.05-123.93	**0.012**
	High	45.65	28.71-62.58		41.41	24.30-58.53	
**Postsurgical concentration**	Low	88.51	71.23-105.79	0.815	85.00	61.21-108.80	0.305
	High	56.72	32.165-81.284		50.90	23.04-78.76	
**Presurgical dimension**	Low	58.47	34.02-82.91	0.155	61.82	35.97-87.67	0.973
	High	49.07	39.48-58.66		49.13	39.92-58.35	
**Postsurgical dimension**	Low	48.35	38.12-58.58	0.616	48.60	38.83-58.37	0.365
	High	93.08	70.72-103.33		78.75	46.54-110.96	

CI, confidential Interval. Significant results are reported in bold.

Figure S1: Correlation between age (years) and A serum exosomes concentration (particles/mL); B exosomal size (nm).

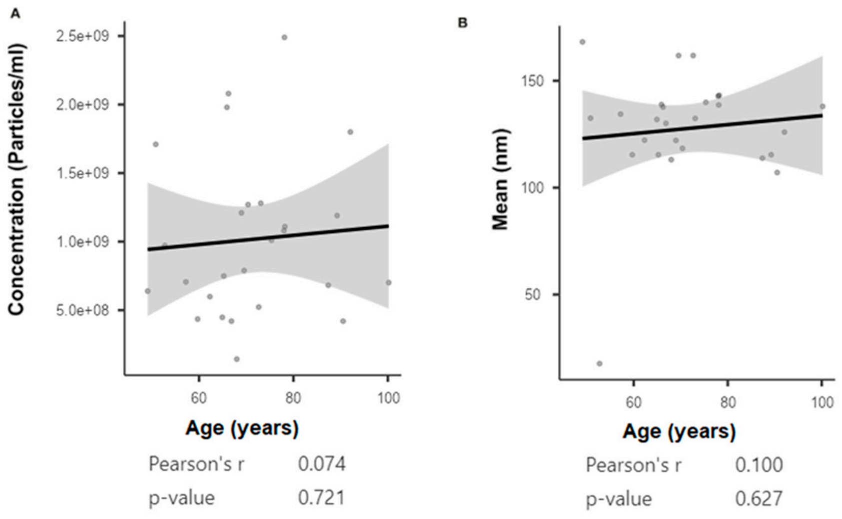

Supplement: Supplementary file 1 [file cancers-15-05693-s001.zip › cancers-2655781-supplementary.pdf]
